# The effects of mung bean and maize intercropping on maize root growth and nitrogen absorption in red soil under different compaction conditions

**DOI:** 10.3389/fpls.2026.1890348

**Published:** 2026-07-13

**Authors:** Xi Yang, Zejun Luo, Jiaxin Xiong, Lu Wang, Xiong Fang, Peng Xiong

**Affiliations:** Key Laboratory of Agricultural Resources and Ecology in Poyang Lake Watershed of Ministry of Agriculture and Rural Affairs in China, College of Land Resource and Environment, Jiangxi Agricultural University, Nanchang, China

**Keywords:** intercropping, nitrogen absorption, root morphology, soil compaction, X-ray computed tomography

## Abstract

Cereal-legume intercropping can promote nutrient absorption by altering crop root morphology. However, its effects on root morphology and nitrogen absorption under compacted soil condition remain unclear. This study aimed to investigate the impact of maize/mung bean intercropping on maize root growth and nitrogen absorption under different soil compaction conditions. Non-compacted (1.2 g/cm³) and compacted (1.5 g/cm³) conditions were established, and three planting patterns were set under each level: maize monoculture, maize and maize intercropping, and maize and mung bean intercropping. X-ray computed tomography (CT) scanning technology and ^15^N isotope labeling method were used to analyze maize root morphology, root-root interactions, and nitrogen absorption. The results showed that regardless of soil compaction, the above-ground biomass of maize in maize/mung bean intercropping was higher than that in maize monoculture and maize/maize intercropping. Maize/mung bean intercropping significantly increased maize root surface area, and also enhanced root volume and total root length compared to maize monoculture under no compaction. Compared with non-compacted condition, soil compaction significantly reduced the root surface area and total root length of maize in maize/mung bean intercropping by 52.32% and 55.92%, respectively, and also significantly decreased the root angle of maize under different treatments. The ^15^N absorption of maize in both monoculture and maize/mung bean intercropping was significantly higher than that in maize/maize intercropping under no compaction, with increases of 120.34% and 122.03%, respectively. Under compacted condition, the ^15^N absorption of maize in maize/mung bean was significantly higher than that in maize monoculture and maize/maize intercropping, increasing by 42.55% and 44.09%, respectively. Our results demonstrate that maize/mung bean intercropping promote maize root growth and improve maize nitrogen absorption even under soil compaction stress. Therefore, maize/mung bean intercropping can serve as a promising planting strategy for enhancing crop productivity in compacted soils.

## Introduction

Soil compaction refers to the process in which soil particles are rearranged under external forces, resulting in reduced porosity and increased bulk density (Gao et al., 2024; Xiong et al., 2022). The destruction of soil structure caused by compaction not only impedes the transport of water and nutrients, reducing nutrient use efficiency in plants, but also restricts ethylene diffusion, which in turn inhibits plant root growth and ultimately leads to yield reduction (Long et al., 2024; Fu et al., 2025; Pandey et al., 2021). Currently, soil compaction has led to global soil degradation, resulting in a 20% - 25% reduction in crop yields. When compaction occurs together with other limiting factors such as drought, yield losses can increase further to 50% - 75%, posing a serious threat to food security (Correa et al., 2019). Studies have shown that the inhibition of root growth and the reduced ability of crops to acquire nutrients from the soil caused by soil compaction are the primary reasons for crop yield losses (Colombi and Keller, 2019; Singh et al., 2021). Therefore, how to effectively enhance crop nutrient absorption capacity and increase crop yields under compacted soil conditions holds significant theoretical value and practical importance for ensuring food security and achieving sustainable agricultural land use.

Intercropping is an important ecological planting pattern in traditional Chinese agriculture, exhibiting significant advantages in improving soil physicochemical properties, modifying crop root morphology and spatial distribution, and enhancing crop nutrient uptake and utilization capacity (Lan et al., 2025; Li et al., 2022; Li et al., 2025a). Compared with monoculture, intercropping systems can improve the efficiency of nutrient resource utilization by crops and enhance the land equivalent ratio (Yu et al., 2015), thereby effectively increasing crop yields (Chen et al., 2026; Weng et al., 2021). Furthermore, intercropping can reduce the application of chemical fertilizer (Yong et al., 2014) and improve fertilizer utilization efficiency. Among all intercropping systems, cereal-legume intercropping is a highly representative planting pattern. Its yield and nutrient uptake advantages stem from both above-ground and underground interspecific interactions, among which root interactions may play an important role (Zheng et al., 2022).

In intercropping systems, changes in the morphological characteristics of crop root systems largely determine their efficiency in utilizing soil nutrients (Zhang et al., 2020). As the primary organs for plants to perceive and respond to changes in the soil environment, roots can absorb and transport soil water and nutrients, playing a key role in the interaction between plants and soil (Kong et al., 2014). Li et al. (2022) found that compared with monoculture maize, intercropping maize with soybean increased maize root length, root surface area, and root volume, and significantly improved nutrient uptake by maize plants. Zheng et al. (2022) reported that the root length density, root surface area density, and nitrogen absorption of maize intercropped with soybean were all significantly higher than those of monoculture maize. They noted that root morphology and spatial distribution determine the nitrogen acquisition capacity of maize in intercropping systems. Therefore, cereal-legume intercropping can effectively enhance the nitrogen uptake and utilization efficiency of crops by changing crop root morphology. In addition to root morphological changes, optimizing field management practices can also significantly improve maize yield and nitrogen partial fertilizer productivity by enhancing soil quality (Zhang et al., 2025).

Currently, most studies on the promotion of efficient nitrogen uptake through cereal-legume intercropping have been conducted under well-structured soil conditions, while the effects of intercropping on crop root morphology and nitrogen uptake in compacted soil remain unclear. To investigate the effects of cereal-legume intercropping on crop root morphology and nitrogen absorption in compacted soil, we conducted a pot experiment using PVC soil columns in a greenhouse. Non-compacted and compacted conditions were established in this experiment, and three cropping systems were set under each level: maize monoculture, maize/maize intercropping, and maize/mung bean intercropping. X-ray computed tomography (CT) scanning and ^15^N isotope labeling techniques were used to analyze root morphological characteristics and nitrogen uptake and accumulation of maize under different soil compaction levels and cropping systems. The aim was to identify the effects of soil compaction and intercropping on maize root growth and nitrogen absorption, providing a theoretical basis for utilizing maize-legume intercropping to increase maize yield in compacted soil. We hypothesized that: (i) maize/mung bean intercropping could promote the growth of maize compared to maize monoculture. (ii) soil compaction inhibited maize root growth, but maize/mung bean intercropping could mitigate the adverse effects of compaction on nitrogen uptake.

## Materials and methods

### Experimental design

The experiment was carried out on August 16, 2025, in the greenhouse at Jiangxi Agricultural University (28°45’26’’N, 115°49’47’’E). The daily average temperature ranged from a minimum of 28.9 °C to a maximum of 42 °C, with relative humidity ranging from 43.8% to 79.6%. The soil was a Quaternary red clay with 34.36% clay (< 0.002 mm), 34.21% silt (0.002 - 0.05 mm), and 31.43% sand (0.05 - 2 mm), and was classified as a clay loam according to the USDA soil taxonomy (Soil Survey Staff, 2003). The soil properties were as follows: pH 4.59, soil organic matter 8.21 g kg^-1^, and total N 0.58 g kg^-1^.

The experimental design was a 2 × 3 completely randomized block. Non-compacted and compacted conditions were established, and three cropping systems were set under each level: maize monoculture, maize and maize intercropping, and maize and mung bean intercropping, resulting in a total of 6 treatments, with 3 replicates for each treatment, for a total of 18 columns. The specific treatments were as follows: Non-compacted maize (1 maize plant per column), non-compacted maize + maize (2 maize plants per column), non-compacted maize + mung bean (1 maize plant and 1 mung bean plant per column), compacted maize (1 maize plant per column), compacted maize + maize (2 maize plants per column), compacted maize + mung bean (1 maize plant and 1 mung bean plant per column).

The air-dried (< 2 mm) soil was filled into PVC columns with a height of 15 cm and an inner diameter of 8.5 cm, to a depth of 14 cm. A bulk density of 1.2 g/cm³ was used to represent the non-compacted treatment, and a bulk density of 1.5 g/cm³ was used to represent the compacted treatment. ^15^N-labeled urea (with a nitrogen content of 46.62%) was mixed evenly with the soil and then filled the columns together. The amount of urea used was 25 mg N/kg, with 0.051g of ^15^N-labeled urea added in the non-compacted treatment and 0.064g of ^15^N-labeled urea added in the compacted treatment. The soil columns were filled layer by layer with air-dried soil. After compacting each layer (10 mm), the soil surface was scraped before the next filling to ensure uniform filling. All soil columns were placed in a water tank and saturated slowly by wetting from the bottom, with water added from the bottom upward. The columns were then drained freely on dry sand until the soil moisture content reached 80% of field capacity.

The maize cultivar used was “Zhengdan 958” and the mung bean cultivar was “Zaolv zhenzhu 2”. Maize and mung bean seeds were germinated on wet gauze at 30 °C for 48 h before sowing. The columns were arranged under natural condition in a completely random pattern. All soil columns were weighed daily and supplemented with distilled water according to water loss to maintain soil moisture levels at 80% field capacity throughout the experimental period. The plants were cultivated in the greenhouse for 12 days. At the end of the experiment on August 28, 2025, the stems and leaves were cut off, and all soil columns were wrapped in plastic film and stored in a refrigerator to prevent soil moisture evaporation and root shriveling, pending CT scanning.

### Measurement indicators and methods

After 12 days of growth, the stems and leaves of the maize plants were removed and dried in an oven at 85°C until they reached a constant weight to determine the above-ground biomass. The dried maize stem and leaf samples were ground using a centrifuge, and the total nitrogen content was determined. The ^15^N abundance was determined by a mass spectrometer (Flash-2000 Delta V ADVANTAGE, USA). The columns were scanned using an industrial X-ray µ-CT scanner in a multiscan mode set at 180 kV and 160 µA, with a pixel/voxel resolution of 80 µm. Each column required approximately 17 minutes and 34 seconds to scan, with a rotation step of 0.48° per second. Projected images were reconstructed using Datos | × 2.0 software (GE, Sensing and Inspection Technologies, GmbH, Wunstorf, Germany), and 16-bit grayscale CT slices were obtained for each column using the filtered back projection algorithm. Continuous two-dimensional slice images were obtained through CT scanning and imported into VGStudio MAX 2025.4 software for 3D reconstruction. The complete root systems were manually extracted to obtain 3D dynamic images of roots using Region Growing tool. The root images were exported from VGStudio MAX 2025.4 software to ImageJ software for preprocessing. Root volume and root surface area were measured using the Particle Analyser tool in ImageJ software. The total root length was obtained using ImageJ software with the Skeletonise 3D and Analyze Skeleton plugins. The root diameter was measured using ImageJ software with the Thickness in BoneJ plugins. Root tortuosity referred to the ratio of actual path length to the shortest possible path length, measured by calculating the ratio of the sum of Branch length to Euclidean distance. Root angle referred to the angle between a straight line passing through two extreme points of a branch and the horizontal plane (Xiong et al., 2020). Root-root interactions referred to the mutual contact and spatial overlap between the root systems of different plants in the soil. For intercropping treatments, root system models of the two crops were extracted separately using VGStudio MAX 2025.4 software. The root-root interaction zones were identified through spatial overlay analysis, and images of these zones exported to ImageJ for calculation.

The calculation methods for nitrogen-related indices are as follows (Chalk et al., 2014):

(1) $$\delta^{15}\text{N}=\frac{15^{\text{N}}{/}^{14}{\text{N}}_{\text{sample}}{-}^{15}\text{N}{/}^{14}{\text{N}}_{\text{standard}}}{15^{\text{N}}{/}^{14}{\text{N}}_{\text{standard}}}\times 1000$$

(2) Atom% 15N= 15NatomsN14atoms+N15atoms × 100

(3) Atom%N15 excess=Atom%N15labeled plant−Atom%N15control plant

(4) Nitrogen accumulation (mg/plant)=Above−ground biomass (g)×Total nitrogen concentration (mg/g)

(5) Above−groundN15 uptake (mg/plant)=Nitrogen accumulation×Atom%N15 excess

### Statistical analysis

Data processing, analysis and graphing for maize above-ground biomass, root morphological characteristics (root volume, root surface area, total root length, and root diameter, etc.), nitrogen uptake and the correlation analysis between maize root morphological characteristics and above-ground nitrogen accumulation were performed using Excel, SPSS 22.0 (SPSS Inc., Chicago, IL, USA) and Origin Pro 2021 9.8 (Origin Lab, Northampton, MA, USA). The significance differences among the treatments were evaluated by one-way ANOVA. The interactions between soil compaction and planting pattern on maize growth and nitrogen uptake were analyzed using two-way ANOVA. Different lowercase letters in the figures indicate significant differences among treatments (*P* < 0.05).

## Results

### Effects of soil compaction and intercropping on maize above-ground biomass

**Figure 1:**
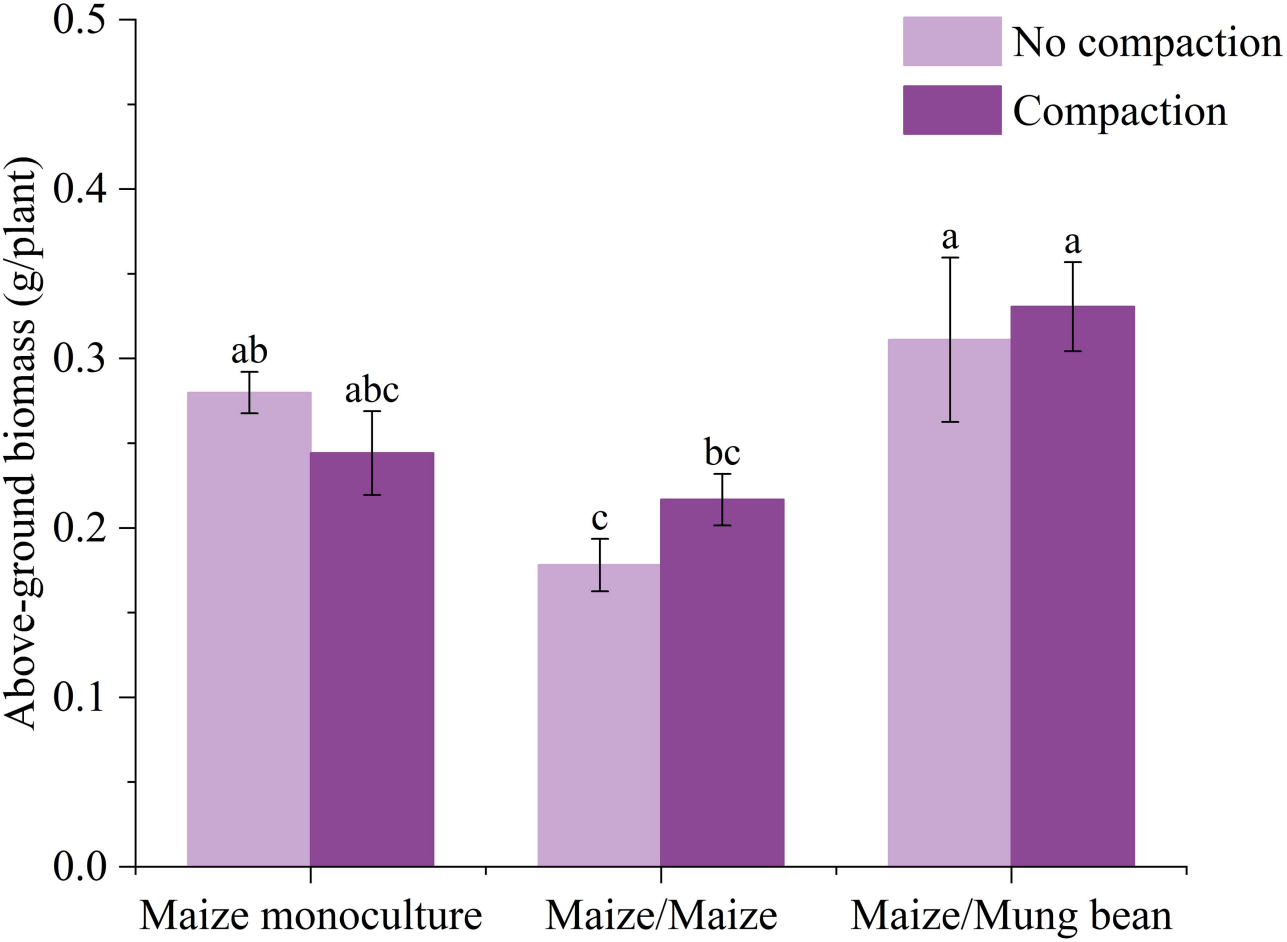
Above-ground biomass of maize under different treatments. Error bars associated with histograms show standard error of the mean (n = 3). Different lowercase letters indicate significant differences among means at *P <* 0.05.

Two-way ANOVA showed that planting pattern significantly affected maize above-ground biomass (**Table 1**). Regardless of soil compaction, the above-ground biomass of maize in maize/mung bean intercropping was the highest (**Figure 1**). Under non-compacted condition, both maize monoculture and maize/mung bean intercropping significantly increased maize above-ground biomass by 57.16% and 74.68% compared with maize/maize intercropping, respectively (*P<* 0.05). However, there was no significant difference in the above-ground biomass of maize between maize monoculture and maize/mung bean intercropping (*P* > 0.05). Compared with maize/maize intercropping, maize above-ground biomass in maize/mung bean intercropping significantly increased by 52.56% under compacted condition (*P* < 0.05). Soil compaction had no significant impact on maize above-ground biomass under three planting patterns (*P* > 0.05).

**Table 1:** Two-way analysis of variance (ANOVA) of maize growth and ^15^N uptake affected by compaction levels and planting pattern.

Variation sources	Above-ground biomass	Root volume	Root surface area	Total root length	Root diameter	Tortuosity	Root angle	^15^N uptake
Compaction levels	0.737	0.068	**0.029**	**0.012**	0.103	0.083	**0.000**	1.000
Planting pattern	**0.002**	0.108	0.107	0.129	0.307	0.761	0.690	**0.002**
Compaction levels × Planting pattern	0.382	0.427	0.330	0.320	0.109	0.131	0.266	**0.032**

The *P* values of the table is shown.

Bold values indicate significant difference (*P* < 0.05).

### Effects of soil compaction and intercropping on maize root morphological characteristics

**Figure 2:**
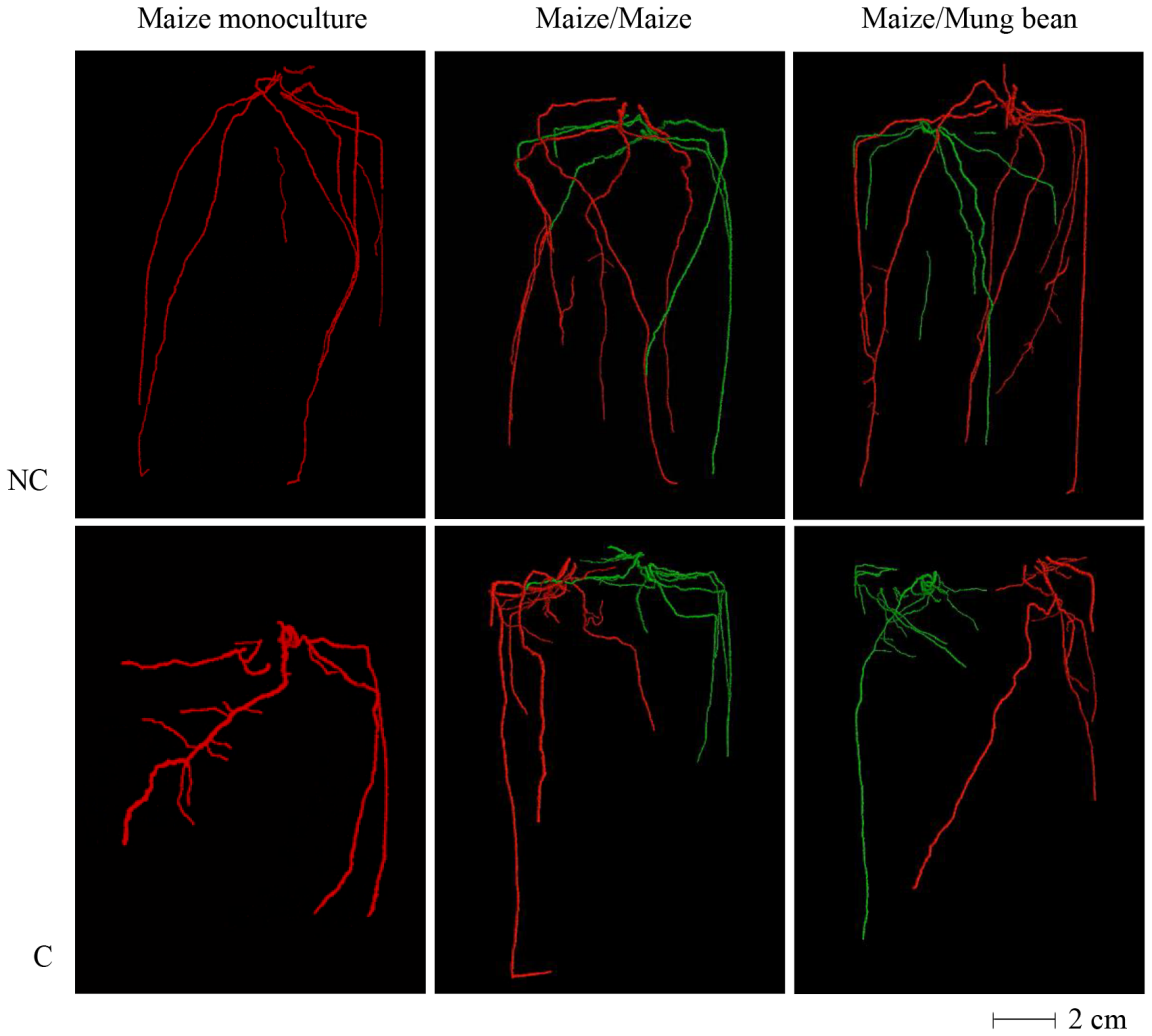
3D root system architecture under different treatments. In the maize/maize intercropping system, both red and green roots represent maize; in the maize/mung bean intercropping system, red roots represent maize and green roots represent mung bean. NC, No compaction; C, Compaction.

**Figure 3:**
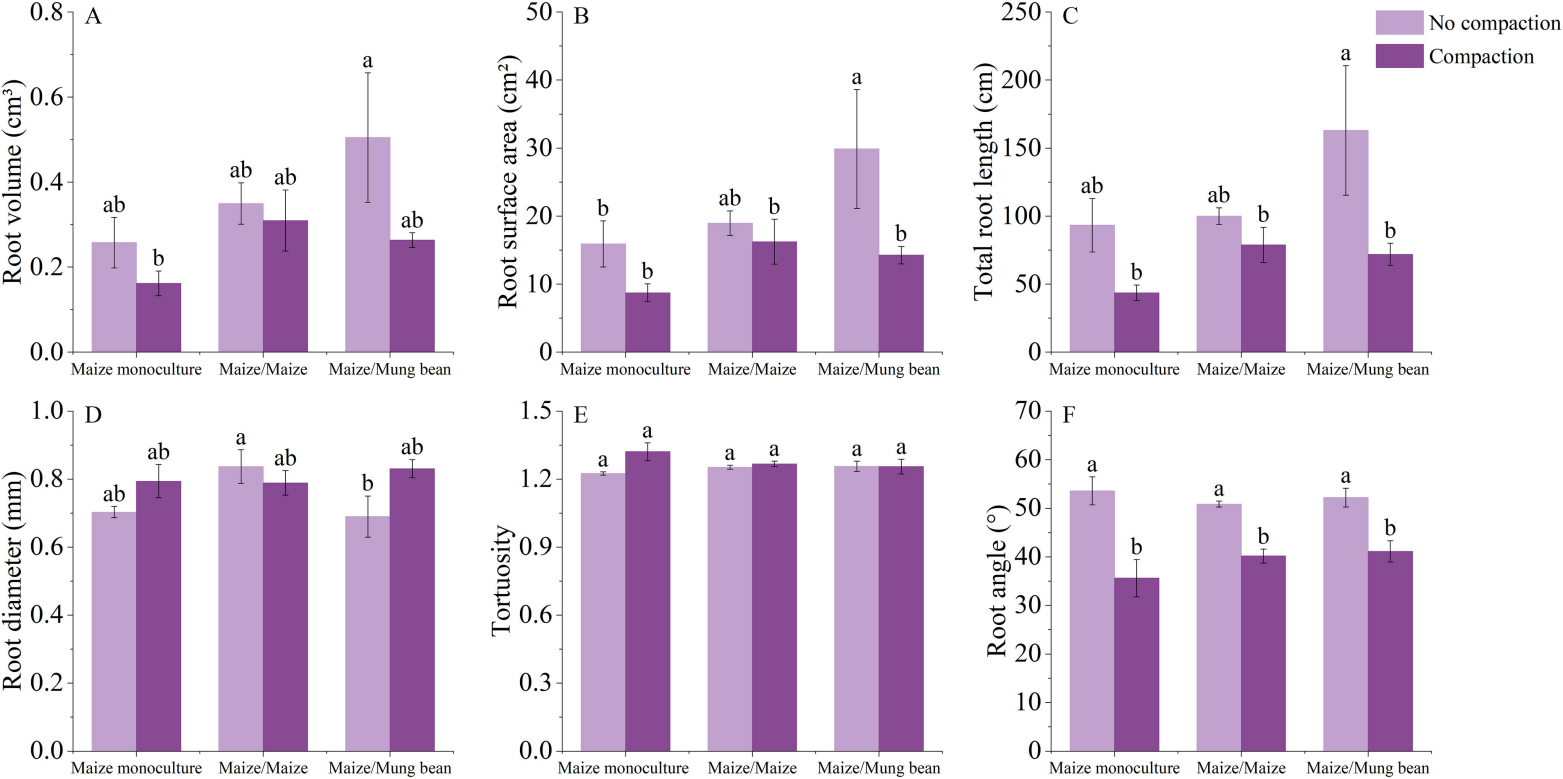
Root architecture characteristics of maize based on X-ray CT images under different treatments. **(A)** Root volume. **(B)** Root surface area. **(C)** Total root length. **(D)** Root diameter. **(E)** Tortuosity. **(F)** Root angle. Error bars associated with histograms show standard error of the mean (n = 3). Different lowercase letters indicate significant differences among means at *P <* 0.05.

The 3D dynamic images of root systems under different treatments showed that soil compaction restricted the growth of maize roots across all treatments, whereas intercropping promoted root growth and improved root morphology (**Figure 2**). There was no significant difference in maize root volume between the treatments of maize monoculture and intercropping, regardless of whether the soil was compacted (*P* > 0.05; **Figure 3A**). The root surface area of maize intercropped with mung bean increased significantly by 87.94% under no compaction compared to maize monoculture (*P* < 0.05; **Figure 3B**). Furthermore, no significant difference was observed among the treatments under compacted condition (*P* > 0.05). Two-way ANOVA showed that soil compaction significantly affected root surface area (**Table 1**). Compared with the non-compacted treatment, soil compaction reduced the root surface area of maize in maize/mung bean intercropping by 52.32% (*P* < 0.05). Regardless of whether the soil was compacted, there was no significant difference in total root length of maize across the different planting patterns (*P* > 0.05; **Figure 3C**). Two-way ANOVA showed that soil compaction significantly affected total root length (**Table 1**). Soil compaction significantly reduced the total root length of maize intercropped with mung bean by 55.92% compared with non-compacted condition (*P* < 0.05), but did not significantly affect monoculture maize or maize/maize intercropping (*P* > 0.05). The root diameter of maize in maize/maize intercropping was significantly greater than that in maize/mung bean intercropping under no compaction, with a 21.3% increase in thickness (*P* < 0.05; **Figure 3D**). No significant difference was observed among the treatments under compacted condition (*P* > 0.05). Furthermore, soil compaction had no significant impact on maize root diameter (*P* > 0.05). Soil compaction and intercropping had no significant impact on the tortuosity of maize roots (*P* > 0.05; **Figure 3E**). Root angle was significantly affected by compaction in all treatments (**Table 1**). Compared with non-compacted treatments, soil compaction resulted in a significant reduction of 33.53% in root angle for monoculture maize (*P* < 0.05; **Figure 3F**), a significant reduction of 21.03% in root angle for maize in maize/maize intercropping (*P* < 0.05), and a significant reduction of 21.18% in root angle for maize intercropped with mung beans (*P* < 0.05).

### Morphological characteristics of root-root interactions under different intercropping treatments

**Figure 4:**
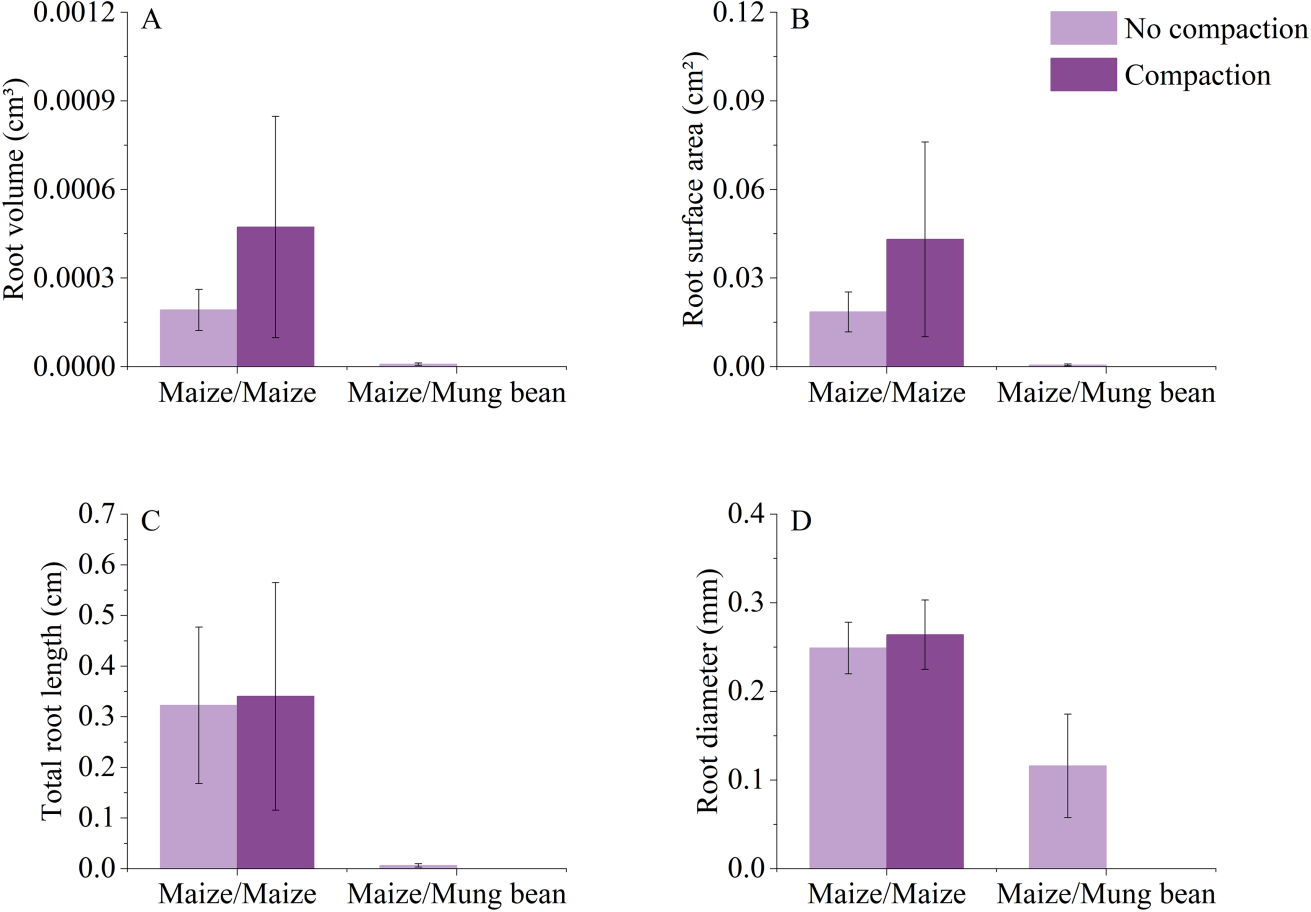
Morphological characteristics of root-root interactions in intercropping systems. **(A)** Root volume. **(B)** Root surface area. **(C)** Total root length. **(D)** Root diameter. Error bars associated with histograms show standard error of the mean (n = 3).

There was no significant difference in interaction root volume, surface area, total root length, or root diameter between maize/maize intercropping and maize/mung bean intercropping regardless of soil compaction (*P* > 0.05; **Figure 4**). Compared with non-compacted condition, soil compaction had no significant effect on the interaction root characteristic in either intercropping system (*P* > 0.05). No root-root interactions occurred in the maize/mung bean intercropping system under compacted soil condition.

### Effects of soil compaction and intercropping on maize ^15^N uptake

Two-way ANOVA results showed that planting pattern had a highly significant effect on maize ^15^N uptake (**Table 1**). The ^15^N uptake in maize monoculture and maize/mung bean intercropping was significantly higher than that in maize/maize intercropping under no compaction, with increases of 120.34% and 122.03%, respectively (*P* < 0.05; **Table 2**). There was no significant difference in ^15^N uptake between maize monoculture and maize/mung bean intercropping (*P* > 0.05). Maize/mung bean intercropping increased maize ^15^N uptake by 42.55% under compacted condition compared to maize monoculture (*P* < 0.05). Compared with maize/maize intercropping, maize/mung bean intercropping increased maize ^15^N uptake by 44.09% (*P* < 0.05). However, there was no significant difference between maize monoculture and maize/maize intercropping (*P* > 0.05). Soil compaction had no significant effect on the ^15^N uptake of maize under different treatments compared with the non-compacted treatment (*P* > 0.05; **Table 1**).

**Table 2:** Effect of intercropping on nitrogen uptake in maize.

Treatment		Atom% ^15^N excess	^15^N uptake (mg/plant)	N accumulation (mg/plant)
Compaction levels	Planting pattern			
No compaction	Maize monoculture	1.5015(0.0564)a	0.130ab	8.70a
	Maize/Maize	1.0099(0.1965)a	0.059c	6.07bc
	Maize/Mung bean	1.5484(0.1083)a	0.131ab	8.42ab
Compaction	Maize monoculture	1.4226(0.0166)a	0.093bc	6.53abc
	Maize/Maize	1.5947(0.0661)a	0.094bc	5.91c
	Maize/Mung bean	1.5417(0.0268)a	0.134a	8.70a

Different lowercase letters indicate significant differences among treatments at *P <* 0.05.

### Correlation between root morphological characteristics and nitrogen accumulation in intercropped maize

**Figure 5:**
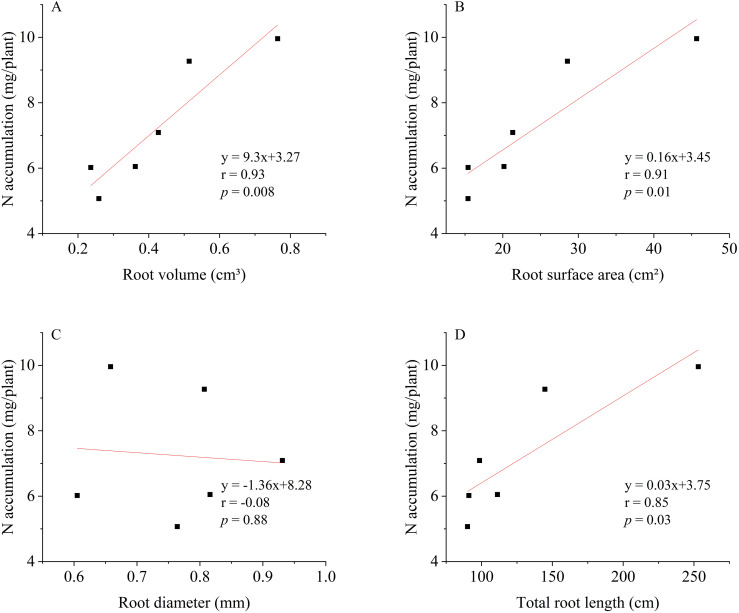
Correlations between root architecture and nitrogen accumulation in maize under intercropping systems without compaction. **(A)** Root volume. **(B)** Root surface area. **(C)** Root diameter. **(D)** Total root length.

**Figure 6:**
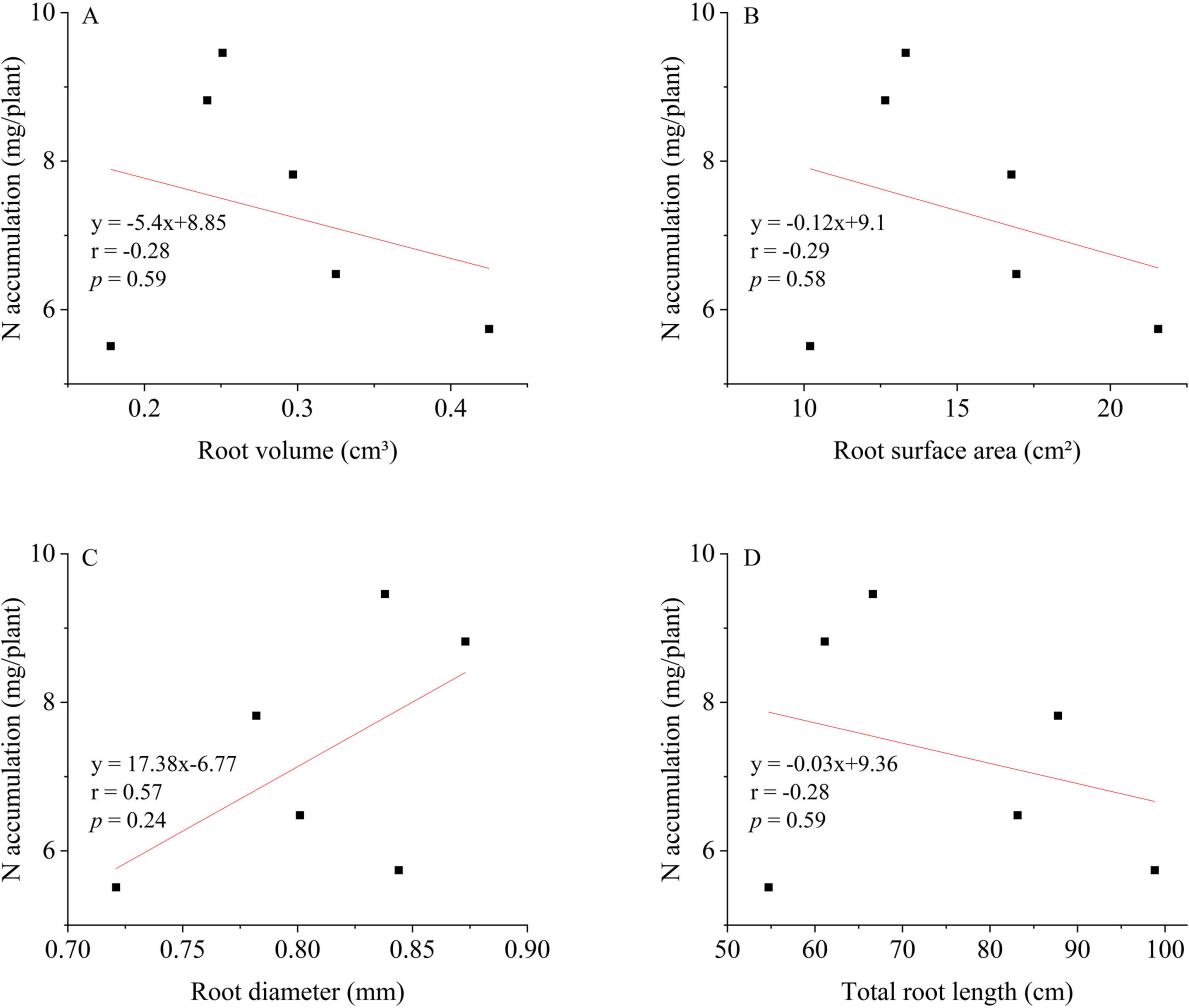
Correlations between root architecture and nitrogen accumulation in maize under intercropping systems with compaction. **(A)** Root volume. **(B)** Root surface area. **(C)** Root diameter. **(D)** Total root length.

Nitrogen accumulation in the above-ground parts of maize showed a highly significant positive correlation with root volume (*P* < 0.01; **Figure 5A**), and a significant positive correlation with root surface area and total root length under non-compacted condition (*P* < 0.05; **Figures 5B, D**). However, there was no significant correlation between root diameter and nitrogen accumulation in the above-ground parts of maize (*P* > 0.05; **Figure 5C**). This indicated that the increase in root volume, root surface area, and total root length of intercropped maize under non-compacted condition can significantly promote nitrogen uptake by maize. Under compacted condition, no significant correlations were observed between root morphological characteristics and above-ground nitrogen accumulation (*P* > 0.05; **Figure 6**).

## Discussion

### Influence of intercropping on maize above-ground biomass under different soil compaction conditions

Maize/mung bean intercropping increased maize above-ground biomass under no compaction compared to maize monoculture (**Figure 1**). This finding was consistent with our first hypothesis. Compared with maize/maize intercropping, both maize monoculture and maize/mung bean intercropping significantly increased maize above-ground biomass. This indicated that intense intraspecific competition between the two maize plants in maize/maize intercropping significantly inhibited their above-ground growth in non-compacted soil. Wang et al. (2018) found that as planting density increased, the biomass of individual maize plants decreased significantly under intercropping cultivation. Yan et al. (2024) reported that although increasing planting density improved population yield, it led to a decrease in individual plant productivity, and high density resulted in a significant decline in individual plant biomass accumulation. Maize/mung bean intercropping promoted maize above-ground growth. Therefore, maize/mung bean intercropping is a planting pattern conducive to the accumulation of maize above-ground biomass under non-compacted condition. Maize/mung bean intercropping increased maize above-ground biomass under compacted condition compared to maize monoculture. Maize/mung bean intercropping significantly increased maize above-ground biomass compared with maize/maize intercropping. This indicated that maize and mung bean intercropping resulted in spatiotemporal niche separation due to their different resource requirements under soil compaction stress (Zhao et al., 2020), thereby reducing interspecific competition and promoting the accumulation of maize above-ground biomass (Zhang et al., 2011). Regarding the effect of soil compaction on maize above-ground biomass, we did not observe significant differences under three planting patterns (**Table 1**). This may be related to our relatively short experimental duration. Previous studies have shown that significant effects of soil compaction on crop above-ground biomass typically require a longer experimental duration to become apparent. Zhu et al. (2024) found that soil compaction had a significant negative effect on above-ground biomass, particularly evident 28 days after sowing. Similarly, Zhang et al. (2023) reported that soil compaction significantly reduced maize above-ground biomass as early as 30 days after sowing. Our experimental period may have been too short to assess the impact of soil compaction on maize above-ground biomass. Therefore, future studies should extend the experimental duration to comprehensively evaluate the long-term effects of compaction on above-ground biomass accumulation in intercropping systems.

### Influence of intercropping on maize root morphology under different soil compaction conditions

Maize/mung bean intercropping enhanced the root volume and total root length of maize under no compaction compared to monoculture maize (**Figures 3A, C**), and significantly increased the root surface area (**Figure 3B**). Yang et al. (2022) found that maize showed greater root morphological plasticity than legumes in intercropping systems, with greater changes in root length density and total root surface compared to monoculture. In our study, maize exhibited greater root morphological plasticity than mung bean under intercropping condition, leading to significant improvements in maize root morphology. This indicated that maize/mung bean intercropping can expand the spatial distribution of roots, and increase the contact area between roots and soil, which was consistent with the findings of Li et al. (2022). Intercropping had no significant effect on the morphological characteristics of maize roots under compacted condition. Soil compaction reduced the root volume, root surface area, and total root length of maize across all treatments compared with the non-compacted treatment. Among these, soil compaction had the greatest impact on maize/mung bean intercropping, resulting in a significant decrease in maize root surface area by 52.32% and total root length by 55.92% (*P* < 0.05; **Figures 3B, C**). This may be due to soil compaction hindering root penetration by increasing soil bulk density and reducing porosity (Correa et al., 2019; Tracy et al., 2011), restricting the distribution range of roots, and ultimately slowing the growth of maize roots (Lipiec et al., 2012; Nosalewicz and Lipiec, 2014). The effect of soil compaction on root angle was very pronounced, with compaction significantly reducing the root angle of maize in monoculture, maize/maize intercropping, and maize/mung bean intercropping (**Figure 3F**). This indicated that maize roots tended to grow more horizontally in compacted soil, possibly because increased mechanical resistance made it difficult for maize roots to penetrate deeper. If the root diameter and angle were insufficient to penetrate a strong soil layer, the roots may grow horizontally as they continued to develop. Studies have found that root angles were relatively gentle in compacted soil in ryegrass (Colombi and Walter, 2016) and lupine plants (Chen et al., 2014). Ramalingam et al. (2017) also found in rice that severe compaction significantly reduced the proportion of deep roots (at angles of 45°-90°), indicating that high compaction intensity forced roots to deflect horizontally by increasing soil mechanical resistance. Notably, the study also found that moderate compaction promoted deep root growth, indicating that moderate compaction actually promoted the growth of deep roots, suggesting that a moderate increase in soil compaction may improve root-soil contact, whereas excessive compaction restricted vertical root penetration due to excessive mechanical resistance. It should be noted that a substantial number of insignificant results for root traits in this experiment may be attributed to the inherent limitations of pot experiments. Compared to field conditions, the confined soil volume in PVC columns restricted root growth space, which may limit the morphological plasticity of roots and reduce the magnitude of differences between treatments. In the future, field studies are needed to confirm our conclusions.

### Influence of intercropping on maize ^15^N absorption under different soil compaction conditions

The ^15^N absorption of maize in maize monoculture and maize/mung bean intercropping was significantly higher than that in maize/maize intercropping with no compaction (**Table 2**). This may be because maize/mung bean intercropping changed the root morphology of maize, enhancing its capacity to absorb nitrogen by increasing root volume and root surface area (Li et al., 2022). In contrast, maize/maize intercropping involved intense intraspecific competition, leading to increased competition for nitrogen between the roots of two maize plants, which resulted in reduced ^15^N absorption by individual maize plant. In compacted soil, the ^15^N absorption of maize in maize/mung bean intercropping was significantly higher than that in maize monoculture and maize/maize intercropping, with increases of 42.55% and 44.09%, respectively (**Table 2**), possibly because mung bean met most of its own nitrogen requirements through symbiotic nitrogen fixation, resulting in lower uptake of soil nitrogen and thereby decreasing direct competition with maize for nitrogen (Wang and Gao, 2020). Moreover, maize had a greater competitive capacity for light, heat, water, and nutrients than mung bean, and this competitive advantage persisted even under soil compaction, making the intercropping system more conducive to maize absorbing more nitrogen (Sun et al., 2024; Raza et al., 2025; Li et al., 2025b). By comparing the two soil compaction conditions, we found that for maize monoculture, compaction reduced its ^15^N absorption. For maize/maize intercropping, compaction increased ^15^N absorption. This may be because soil compaction had the least impact on the root growth of maize in the intercropping system, and promoted root-root interactions. The roots of the two maize plants had more contact within the limited space, which may enable them to jointly explore more soil pores and acquire more nitrogen. Two-way ANOVA revealed the interactive effects of soil compaction and planting pattern on ^15^N uptake (*P* = 0.032; **Table 1**). Jones et al. (2004) noted that under stress conditions such as hypoxia, the rate of root exudation can increase significantly due to loss of membrane integrity or a breakdown in normal cell metabolism. In this study, hypoxia induced by soil compaction may have stimulated the release of root exudates from maize and mung bean. Under compacted condition, maize/mung bean intercropping may have enriched beneficial rhizosphere microorganisms (such as Bradyrhizobium) through root exudates (Dong et al., 2022). Dong et al. (2024) further demonstrated that in a maize/peanut intercropping system, Bradyrhizobium could indirectly influence the efficiency of nitrogen fixation and transfer to maize, thereby enhancing nitrogen acquisition by maize. This mechanism may have enabled maize to maintain high ^15^N absorption despite a significant reduction in root surface area and total root length.

### Relationship between maize root morphological characteristics and nitrogen accumulation

The root volume, root surface area, and total root length of intercropped maize all showed a significant positive correlation with above-ground nitrogen accumulation without compaction (**Figure 5**). This indicated that increases in root volume, root surface area, and total root length were important factors promoting nitrogen absorption by intercropped maize in maize/mung bean intercropping system. Previous studies have shown that root morphological parameters in maize and rice were significantly correlated with nitrogen uptake (Li et al., 2022; Qu et al., 2016; Chen et al., 2017). Our result was consistent with these findings, suggesting that root morphology can serve as an important indicator for evaluating maize nitrogen uptake. However, the correlations between all root morphological indices (root volume, root surface area, total root length, and root diameter) and nitrogen accumulation were not significant under compacted condition (**Figure 6**). Although soil compaction inhibited maize root growth, leading to a significant decrease in root surface area and total root length of maize intercropped with mung bean, the nitrogen absorption advantage of maize did not disappear due to the suppression of root morphology. This finding was consistent with our second hypothesis. This may be related to interspecific interactions improving the soil microenvironment (Chen et al., 2025). The differences in correlations between non-compacted and compacted conditions indicated that the key factors affecting nitrogen accumulation varied with soil environment. Under non-compacted condition, the expansion of root morphology was the primary factor promoting nitrogen accumulation; whereas under compacted condition, this morphological advantage disappeared, but nitrogen uptake was still maintained through other mechanisms. Nitrogen accumulation of maize in maize/mung bean intercropping (8.70 mg/plant) was higher than in maize monoculture (6.53 mg/plant) and maize/maize intercropping (5.91 mg/plant) under compacted condition (**Table 2**), despite significant reductions in both root surface area and total root length. A comparison of the two compaction treatments revealed that compaction reduced nitrogen accumulation in maize monoculture and maize/maize intercropping, but had no negative effect on maize/mung bean intercropping, indicating that maize/mung bean intercropping could mitigate the adverse effects of compaction on nitrogen uptake. Numerous studies have demonstrated that plants can actively recruit beneficial rhizosphere microorganisms through root exudates to mitigate abiotic stress (Ahlawat et al., 2024). Zhang et al. (2023) noted that in compacted silt loam soil, maize secreted organic acid anions and recruited beneficial actinomycetes to enhance phosphorus uptake; Parasar et al. (2024) found that under stress conditions, plants released root exudates with different blends to alleviate stress by modulating the rhizosphere microbiome. Furthermore, Wang et al. (2024) reported that intercropping systems can promote soil nitrogen transformation and maize nitrogen uptake by increasing the abundance of functional microorganisms involved in ammonia oxidation, denitrification, and anammox in the rhizosphere soil, as well as the expression of related genes. Therefore, maize/mung bean intercropping may enable maize to maintain high nitrogen uptake in compacted soils even under conditions of suppressed root growth through similar microbial regulatory mechanisms.

## Conclusions

Regardless of soil compaction, intercropping maize with mung bean is beneficial to the accumulation of above-ground biomass in maize. Maize/mung bean intercropping altered maize root morphology by increasing root surface area, root volume, and total root length in non-compacted soils. Intercropping had no significant effect on the morphological characteristics of maize roots under compaction condition. Soil compaction significantly reduced the root angle of maize in all treatments, hindering the vertical penetration of roots. Correlation analysis showed that the root volume, root surface area, and total root length of intercropped maize were significantly positively correlated with above-ground nitrogen accumulation under no compaction, indicating that the expansion of root morphology was an important factor in promoting nitrogen absorption. However, maize intercropped with mung bean still maintained high nitrogen absorption under compaction condition despite suppressed root growth. Thus, maize/mung bean intercropping is a planting pattern with potential for application, particularly in compacted soils.

## Data Availability

The original contributions presented in the study are included in the article/supplementary material. Further inquiries can be directed to the corresponding author.
